# Effect of Four Approaches to Oral Feeding Progression on Clinical Outcomes in Preterm Infants

**DOI:** 10.1155/2015/716828

**Published:** 2015-04-27

**Authors:** Rita H. Pickler, Barbara A. Reyna, Paul A. Wetzel, Mary Lewis

**Affiliations:** ^1^Nursing Cincinnati Children's Hospital Medical Center, 3333 Burnet Avenue, MLC 11016, Cincinnati, OH 45229, USA; ^2^Neonatal Nurse Practitioner, Children's Hospital of Richmond at VCU, P.O. Box 985912, Richmond, VA 23298, USA; ^3^Department of Biomedical Engineering, School of Engineering, Virginia Commonwealth University, P.O. Box 843067, Richmond, VA 23284-3067, USA; ^4^Children's Hospital of Richmond at VCU, P.O. Box 985912, Richmond, VA 23298, USA

## Abstract

*Background*. The purpose of this study of preterm infants was to test the effect of four approaches to the time of transition from gavage to full oral feedings, time to discharge, and weight gain during the transition. *Methods*. A randomized experimental design was used with four intervention groups: early start (32 weeks' postmenstrual age)/slow progressing experience (gradually increasing oral feedings offered per day); early start/maximum experience (oral feedings offered at every feeding opportunity); late start (34 weeks' postmenstrual age)/slow progressing experience; and late start/maximum experience. *Results*. The analysis included 86 preterm infants. Once oral feedings were initiated, infants in the late start/maximum experience group achieved full oral feeding and were discharged to home significantly sooner than infants in either early start group. Although not significantly different, these infants also achieved these outcomes sooner than infants in the late start/slow progressing experience group. There were no differences in weight gain across groups. *Conclusions*. Results suggest starting oral feedings later in preterm infants may result in more rapid transition to full oral feedings and discharge although not at early postnatal ages. Provision of a more consistent approach to oral feeding may support infant neurodevelopment and reduce length of hospitalization.

## 1. Introduction

Although the preterm birth rate has been declined over the last several years, it continues to hover around 11% [1]. Moreover, despite improved survival of preterm infants and major changes in their care, many common caregiving issues remain, including when to initiate and when and how to advance oral feedings to achieve the best outcomes [2]. Thus, the transition from gavage to oral feedings remains a clinical challenge for both infants and their caregivers. Successful transition to oral feeding is important as competence at oral feeding is a criterion for hospital discharge [3]. Delay in achieving competence at oral feeding is one of the major reasons for delays in hospital discharge for otherwise physiologically stable preterm infants [4–6].

Achieving oral feeding competence takes time, with the transition from gavage to all oral feedings reportedly taking from 10 to 14 days [7]. Although breast feeding may present fewer physiologic challenges than bottle feeding for the preterm infant, most preterm infants are bottle fed (formula or expressed breast milk) at least some of the time while being in the hospital [8]. Thus, management of oral feedings for preterm infants is a key aspect of hospital care. Currently, there are few evidence-based protocols to guide clinicians as they assist infants in achieving competence at oral feeding and those that exist are often not based soundly on research evidence. Consequently, already vulnerable infants are subject to a trial-and-error approach to this most complex and critical life-sustaining activity, with potentially harmful short- and long-term effects.

Successfully making the transition from gavage to oral feedings requires the infant to coordinate suck-swallow-breathe and maintain autonomic nervous system organization. Competence at oral feeding requires the infant to consume a prescribed volume of formula or breast milk and do so in an efficient manner without undue physiological costs (i.e., apnea, bradycardia). Further, there is increasing evidence that both the quantity and the quality of oral feeding experience may play a role in the feeding transition [5–10]. Infant's medical condition and neurological maturity also influence the transition [2, 10, 11]. We also examined the effect of infant morbidity on these clinical outcomes.

The purpose of this study was to prospectively test four approaches to the transition from gavage to oral feedings in preterm infants. This paper specifically presents the analysis of our aim to determine the effect of the four feeding approaches on clinical outcomes (time to full oral feedings from the start of oral feeding, time to discharge from the start of oral feeding, and weight gain during the transition) in preterm infants.

## 2. Materials and Methods

A randomized experimental design was used to test four different approaches to the transition to full oral feedings. Within morbidity strata infants were randomly assigned to one of four feeding approaches: (1) early start/slow progressing experience, (2) early start/maximum experience, (3) late start/slow progressing experience, and (4) late start/maximum experience. Infants in the early start groups began the transition from gavage to full oral feedings at 32 weeks' postmenstrual age (PMA) while infants in the late start groups began the transition to full oral feeding at 34 weeks' PMA. In the slow progressing experience groups, the number of oral feedings offered daily was gradually increased over a 14-day period from 2 per day to 8 per day. In the maximum experience groups, 8 of 8 feedings were offered orally per day throughout the 14-day protocol. These feeding approaches were based on predicted patterns of transition found in earlier studies [5, 9]. We hypothesized that infants who were started later on oral feedings and offered maximum experience at oral feeding would have significantly shorter transition times from gavage to full oral feeding and to discharge. We further hypothesized that there would be no difference in weight gain among infants in the four feeding approaches.

### 2.1. Ethics Review

The study was approved by an institutional review board and all parents of participating infants gave informed written consent.

### 2.2. Sample

A convenience sample was recruited from a 34-bed, Level III, neonatal intensive care unit (NICU). Infants were included in the sample if (1) infant's gestational age at birth was less than 32 weeks, (2) the infant was receiving enteral feedings every three hours, (3) the infant was able medically to feed orally by 32 weeks' PMA, and (4) the parents gave consent for infant's participation. Infants were excluded if (1) they were unable to begin oral feeding at 32 weeks' PMA due to gastrointestinal, craniofacial, cardiovascular, neuromuscular, and/or genetic defects, (2) they had surgical necrotizing enterocolitis, or (3) they needed ventilator support, including nasal continuous positive airway pressure (CPAP), after 32 weeks' PMA. Infants receiving low flow oxygen by cannula were included.

### 2.3. Intervention Feeding Approaches

The feeding approaches were developed from previous research about the effect of maturity and experience on the development of oral feeding skills in preterm infants. As seen in Table 1, each approach was a 14-day protocol with two levels of maturity at the start of oral feeding and two levels of feeding experience throughout the 14 days. Infants were fed every 3 hours (8 feedings a day) consistent with unit practice. Infants were not expected to consume all prescribed fluid orally and the number of feedings offered orally was not contingent on how successful they had been at consuming the volume offered at previous oral feedings. The amount consumed orally at each feeding was measured and any formula remaining after the oral feeding attempt was delivered by gavage. Infants were fed with either formula or breast milk; mothers fed their infant with breast at any feeding they desired. At all feedings, parents were encouraged to feed their infants. The four feeding approaches were all 14-day protocols; all infants were offered 8 oral feedings per day after day 14 and until discharge.

It is important to note that these were offerings of oral feedings; we did not expect infants to consume 100% of prescribed formula orally at every offered oral feeding. We were aware that even on the slow progressing approaches some infants would be too sleepy or not alert enough to feed orally. We recorded reasons why infants were not fed orally (not awake, physiologically unstable, other reasons not related to infant well-being) and why they did not finish feedings, oral feedings.

### 2.4. Procedures

**Figure 1:**
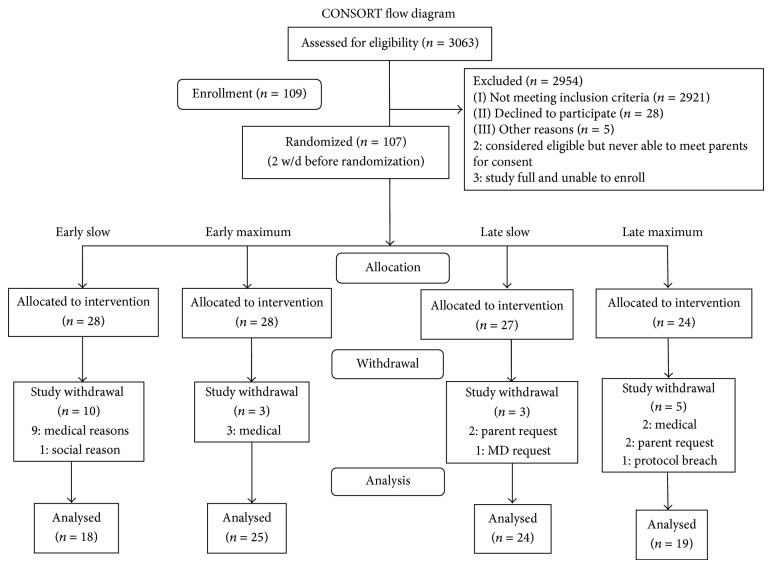


A convenience sample was recruited. All infants admitted to the NICU were screened for eligibility and followed until they were between 30 and 32 weeks' PMA; they were recruited to the study at that time if they continued to meet eligibility. Parents were approached by the study's clinical coordinator who was trained to obtain consents. Once consented, infants' medical histories were reviewed and a morbidity score was assigned using Neonatal Medical Index (NMI) [12]. NMI classifications range from 1 for the healthiest infants to 5 for the most ill. Infants were randomly assigned using stratification by NMI to one of four groups. Infants with a NMI classification of 1 or 2 (healthiest) were stratified together since infants in these groups performed similarly on outcomes in our previous research and infants with a NMI 4 or NMI 5 classification (most ill) were stratified together as these infants showed the greatest variability on outcomes measures in our previous research [10]. Each feeding approach group was assigned equal numbers of infants, but because 50% of the sample was expected to be classified as NMI 3, we planned for those infants to be overrepresented in the groups with approximately equal numbers in NMI classifications 1 or 2, 4, and 5. The order of assignment to groups was determined by random number generator programmed by the statistician as recommended by Consolidated Standards of Reporting Trials (CONSORT) guidelines [13]. Prior to the start of the study, cards were marked with the assigned group and put in sealed envelopes, which were kept in a locked cabinet in the study office. At the time of enrollment, the next group assignment card for infant's calculated NMI was pulled and the infant was assigned to that group. The study's clinical coordinator entered the plan for starting and progressing oral feedings into the infant's computerized medical care plan, which was used to direct caregiving. See Figure 1 for the study's CONSORT chart.

### 2.5. Outcome Measures

#### 2.5.1. Time to Full Oral Feedings

Time to full oral feeding was defined as the length of time (in days) between the initiation of oral feeding and the date of full oral feeding. Full oral feedings were defined by unit practice as the point at which the infant was consuming all prescribed volume of formula/breast milk orally for 24 hours.

#### 2.5.2. Time to Discharge

Time to discharge was defined as the length of time (in days) between the date of first oral feeding and the discharge date.

#### 2.5.3. Weight Gain during the Transition

Average weekly weight gain was included as an outcome of interest since failure to gain weight adequately was a concern for infants in the early start and/or maximum experience groups. Infant weight was recorded daily by the study team.

### 2.6. Statistical Methods

Prior to conducting the analysis, the treatment groups were compared using either a chi-square or one-way ANOVA, depending upon the nature of the variable of interest. All analyses were performed using SAS software (SAS version 9.2, JMP version 8.0.2, SAS Institute Inc., Cary, NC). The primary outcomes, time to full oral feedings and time to discharge from the start of oral feedings, were log transformed because of skewness. Average weight gain per week was computed by performing a linear regression separately for each infant. The analysis included infants who completed the prescribed intervention; that is, these infants completed their assigned 14-day feeding approach as well as infants who attained full oral feeding and were discharged to home before the 14th day of the protocol.

## 3. Results

The final sample consisted of 86 infants; 44 were male and the median birth weight was 1382 grams (range = 590 to 2465). Sixty-eight (68) infants were Black/African American and 13 were White, 3 were of more than one race, and 2 did not have race indicated; 5 infants were Hispanic. Morbidity was measured by NMI; there were 19 infants classified as NMI 1, 16 at NMI 2, 30 at NMI 3, 8 at NMI 4, and 13 at NMI 5 groups. As seen in Table 2, there were no differences in the gender, race, ethnicity, birth gestation, birth weight, or morbidity across the four feeding approach groups.

On average, infants in the study achieved full oral feedings 14.5 days after starting oral feeding (SD = 9, range = 46 days; PMA = 35.1 weeks, SD = 1.3, range = 6.7 weeks). Infants in the study went home on average 21.3 days after starting oral feedings (SD = 10.2 days, range = 47 days; PMA = 36.1 weeks, SD = 1.4, range = 6.1 weeks).

As seen in Table 3, there were differences in the intervention groups on days to achieve full oral feeding once oral feedings were started (*F* = 6.75, *p* = 0.004). Using the Tukey method for comparison of groups, groups 1 and 2 (32-week start groups) achieved full oral feeding at a mean of 18 days, which was significantly different from group 4 (34-week start, maximum experience) whose time to full oral feeding was 9 days from the start of oral feedings (*p* = 0.002 and *p* = 0.001, resp.). Groups 1 and 2 did not differ significantly from group 3, whose mean time to full oral feeding was 14 days, and group 3 did not differ significantly from group 4. The groups also differed significantly on days to discharge from the start of oral feeding (*F* = 6.44, *p* = 0.001). Using the Tukey method for comparison of groups, groups 1 and 2 were discharged to home, at a mean of 26 and 25 days, respectively, following the start of oral feedings, which was significantly longer than infants in group 4 whose time to discharge was 15 days from the start of oral feeding (*p* = 0.003 and *p* = 0.009, resp.). Group 1 was also significantly different from group 3, whose mean time to discharge was 19 days (*p* = 0.03). Group 2 did not differ significantly from group 3 and group 3 did not differ significantly from group 4. As Table 3 also shows, group 3 differed significantly from groups 1 and 2 in PMA at full oral feeding. There were no other significant differences in PMA at full oral feedings or discharge.

On average, all infants gained about 205 grams per week. Weight gain did not vary by group assignment although it did differ by morbidity (*p* = 0.05). Tukey's HSD indicated that infants with a NMI classification of 4 gained weight more slowly than those with a NMI of 3 (Table 4). There were no other differences.

We conducted additional analyses to determine if other variables of potential interest affected the outcomes. We were particularly interested in whether outcomes differed by morbidity, both within groups and overall. When run independent of group assignment, time to full oral feeding and time to discharge from the start of oral feeding both differed significantly by morbidity. Infants at NMI 5 took significantly longer to achieve full oral feeding from the start of oral feeding than infants with a NMI of 1 or 2 (*F* = 4.15, *p* = 0.004). Infants at NMIs 4 and 5 also took significantly longer to be discharged to home following the start of full oral feedings than infants at NMIs 1 and 2 (*F* = 8.32, *p* < 0.000).

Further examination of morbidity differences within groups showed that infants with a NMI of 2 in groups 1 and 2 took significantly longer to achieve full oral feeding than infants of the same NMI in group 4 (*F* = 9.57, *p* = 0.001). Infants with a NMI of 3, the largest and most variable NMI classification, in group 2 also took significantly longer to achieve full oral feedings than infants with the same NMI in group 4 (*F* = 3.14, *p* = 0.046). There were no other significant differences in the time to full oral feeding within groups by NMI classification. Infants with a NMI of 1 in groups 1 and 2 and infants with a NMI of 2 in group 1 took significantly longer to be discharged following the start of oral feedings than infants of the same NMI in group 4 (*F* = 6.22, *p* = 0.004 and *F* = 3.71, *p* = 0.035, resp.). For the infants with a NMI of 3 and the sicker infants at NMIs 4 and 5 there were no significant differences in the time to discharge from the start of oral feedings regardless of group assignment.

A simple regression analysis of time to full oral feeding with stepwise loading of potential variables of interest (birthweight, birth gestation, morbidity, sex, race, and group assignment) revealed a *R* ^2^ of 0.40 (*F* = 3.45, *p* < 0.000) with only group assignment contributing significantly to the variation. A second regression analysis of time to discharge from the start of oral feeding using the same variables of interest revealed a *R* ^2^ of 0.51 (*F* = 5.24, *p* < 0.000) again with only group assignment contributing significantly to the variation.

## 4. Discussion

The purpose of this study was to prospectively test four approaches to the transition from gavage to oral feedings on clinical outcomes in preterm infants. We hypothesized that infants who were offered maximum experience at oral feeding would have significantly shorter transition times from the start of oral feeding to full oral feeding and to discharge. We further hypothesized that there would be no difference in weight gain among infants in the four feeding approaches.

Our analysis supported our hypotheses. Infants in group 4, who started oral feeding at 34 weeks PMA and received maximum oral feeding experience, after initiating oral feedings, reached full oral feeding 9 days sooner and were discharged to home 9 to 10 days sooner than infants in either group that started at 32 weeks' PMA. Moreover, although not significantly different from infants in group 3 who started oral feeding at 34 weeks' PMA and progressed slowly in oral feeding, infants in the late start/maximum experience group also achieved full oral feeding 5 days sooner and were discharged to home 4 days sooner. Additionally, although offering preterm infants maximum oral feeding experience may raise concerns that greater energy will be expended and result in slower weight gain, we did not observe any difference in weight gain across groups.

The transition from gavage feeding to full oral feeding has been studied by numerous researchers who have generally concluded that the transition is influenced by many factors, including infant characteristics and medical complications [10, 14–16]. In this study, we found that older preterm infants at the start of oral feeding made a more rapid transition but only within the context of the assigned feeding approach group. We had a similar finding in regard to infant morbidity; when examined independently, increased morbidity was associated with longer transition; however, these differences were not retained within the context of the feeding approach assignment. Thus, although the transition from the start of oral feeding to full oral feeding and discharge was shorter for the late starting groups, all infants were approximately of the same PMA at both points.

There are also numerous published protocols for progression of nipple feeding frequency [17–21]. However, there is little evidence for the effectiveness of these protocols, including those developed from “cue-based” feeding or “demand/semidemand” feeding approach that continue to advocate for a limited prescribed number of oral feedings based per day that are progressed slowly over the course of many days. This situation is potentially dangerous; the result of a lack of empirically tested protocols means that there is continued use of trial-and-error approaches to oral feeding that fail to take advantage of the human infant's basic need for a consistent approach to feeding [22, 23]. Although the contribution of feeding experience to normal development has been infrequently studied in preterm infants, these infants may fail to develop nutritive sucking skills or they may develop inappropriate oral feeding responses without ample opportunity to practice. Moreover, there is emerging evidence that when infants are not offered oral feedings when they are awake and physiologically stable, there is a prolonged attainment of full oral feeding and a subsequent delay in discharge to home [6], both of which may be predictors of later poor development.

## 5. Conclusions

Based on the study results, we would recommend that nurses and others responsible for managing the oral feeding care of preterm infants consider delaying the start of oral feeding until infants are at least 34 weeks' PMA; there does not appear to be an advantage to starting oral feedings early. Additionally, based on our own prior study results as well as the results of other studies, we would recommend that the start of oral feedings occurs when infants are showing signs of physiological and behavioral readiness, physiologic stability, wakefulness at feeding times, and interest and ability to suck on a nipple. We further recommend that once oral feedings are started, infants be assessed for readiness at every scheduled feeding including observation of physiologic stability and wakefulness or ease with which the infant achieves a stable awake state when handled. If the infant is awake at a scheduled feeding time and is not physiologically distressed, the infant should be offered the opportunity to feed orally. We recognize that these recommendations may be at odds with typical NICU practices that advocate for slow progression of the number of oral feedings, often with a prescribed number of oral feedings a day. However, our data clearly show that the opportunity to feed orally, regardless of the volume taken orally, speeds the transition to full oral feeding and to hospital discharge. Moreover, our data clearly indicate that these recommendations may be as appropriate for infants who have high morbidity as they are for infants at lower morbidity. The reduction in costs, to the infant, the family, and the health care system, makes these recommendations well worth the effort to incorporate into practice.

## Figures and Tables

**Table 1:** Description of four feeding approaches.

Approach name	PMA at oral feeding start	Manner of oral feeding progression
Group 1, early start/slow progressing experience	32 weeks	Offered 2 oral feedings per day for 3 days (days 1–3). Oral feedings offered per day increased by 1 feeding every other day until day 14 when offerings were 8 oral feedings each day (days 4-5, 3 oral feeds offered; days 6-7, 4 oral feeds offered; days 8-9, 5 oral feeds offered; days 10-11, 6 oral feeds offered; days 12-13, 7 oral feeds offered; day 14, 8 oral feeds offered).

Group 2, early start/maximum experience	32 weeks	Offered 8 oral feedings every day, at each of 8 scheduled daily feedings starting on day 1.

Group 3, late start/slow progressing experience	34 weeks	Offered 2 oral feedings per day for 3 days (days 1–3). Oral feedings offered per day increased by 1 feeding every other day until day 14 when offerings were 8 oral feedings each day (days 4-5, 3 oral feeds offered; days 6-7, 4 oral feeds offered; days 8-9, 5 oral feeds offered; days 10-11, 6 oral feeds offered; days 12-13, 7 oral feeds offered; day 14, 8 oral feeds offered).

Group 4, late start/maximum experience	34 weeks	Offered 8 oral feedings every day, at each of 8 scheduled daily feedings starting on day 1.

**Table 2:** Participant characteristics.

	Feeding approach (intervention) groups			
	Group 1: 32 slow (*n* = 18)	Group 2: 32 max (*n* = 25)	Group 3: 34 slow (*n* = 24)	Group 4: 34 max (*n* = 19)
Gender				
Female	10	11	10	11
Male	8	14	14	8
Race				
Black	15	19	18	16
White	2	4	5	2
>1 race	0	1	1	1
Not reported	1	1	0	0
Mean Birthweight in grams (SD; min–max)	1349 (404; 700–1970)	1447 (392; 630–2465)	1355 (382; 590–1930)	1362 (282; 880–1700)
Mean birth gestation in weeks (SD; min–max)	29.5 (2.5; 24–32)	29.9 (2.2; 24–32)	29.9 (2.2; 24–32)	29.9 (2.1; 25–32)
Morbidity				
NMI Classification				
1	4	6	7	5
2	5	4	6	4
3	4	10	6	6
4	2	2	1	2
5	3	3	4	2

**Table 3:** PMA and time in days to full oral feeding and discharge from first oral feeding.

	Intervention groups			
	Group 1: 32 slow (*n* = 18)	Group 2: 32 max (*n* = 25)	Group 3: 34 slow (*n* = 24)	Group 4: 34 max (*n* = 19)
Mean time in days from 1st oral feeding to full oral feedings (SD; min–max)	17.8 (1.9; 14.0–21.6)	17.8 (1.6; 14.6–21.0)	14.1 (1.9; 10.8–17.4)	8.8^2^ (1.9; 5.1–12.4)

Mean PMA in weeks at full oral feeding (SD; min–max)	34.8 (0.286; 34.2–35.4)	34.8 (0.243; 34.3–35.3)	36.0^1^ (0.253; 35.4–36.4)	35.1 (0.284; 34.6–35.7)

Mean time in days from 1st oral feeding to discharge (SD; min–max)	25.7 (2.2; 21.2–30.1)	24.8 (1.9; 21.1–28.6)	19.3^3^ (1.9; 15.4–23.1)	15.1^2^ (2.2; 10.8–19.4)

PMA in weeks at discharge (SD; min–max)	35.9 (0.337; 35.3–36.6)	35.8 (0.286; 35.3–36.4)	36.7 (0.286; 36.1–37.2)	36.0 (0.329; 35.4–36.7)

^1^Significantly different from Groups 1 and 2 at *p* < 0.05.

^2^Significantly different from Groups 1 and 2 at *p* < 0.01.

^3^Significantly different from Group 1 at *p* = 0.03.

**Table 4:** Weight gain during oral feedings.

Groups	*N*		Weight gain per week (grams)			
	Infants	Observations	LS mean	SE	95% CI	
Intervention						
Group 1	18	467	202.5	9.3	184.1	221.0
Group 2	25	627	206.0	8.1	190.0	222.1
Group 3	24	446	206.1	8.1	189.9	222.4
Group 4	19	297	207.1	9.1	188.9	225.3
Morbidity						
NMI 1	22	290	214.5	8.9	196.7	232.3
NMI 2	19	279	215.2	9.7	195.9	234.5
NMI 3	26	659	214.7	7.1	200.5	228.9
NMI 4	7	224	169.0^1^	13.7	141.8	196.3
NMI 5	12	385	213.6	10.8	192.3	235.2

^1^Significantly different from NMI 3 at *p* = 0.05.

## References

[1] National Center for Health Statistics (2014). *Final Natality Data*.

[2] White-Traut R., Pham T., Rankin K., Norr K., Shapiro N., Yoder J. (2013). Exploring factors related to oral feeding progression in premature infants. *Advances in Neonatal Care*.

[3] American Academy of Pediatrics (2008). Hospital discharge of the high-risk neonate. *Pediatrics*.

[4] Jadcherla S. R., Wang M., Vijayapal A. S., Leuthner S. R. (2010). Impact of prematurity and co-morbidities on feeding milestones in neonates: a retrospective study. *Journal of Perinatology*.

[5] Pickler R. H., Best A. M., Crosson D. D. (2009). The effect of feeding experience on clinical outcomes in preterm infants. *Journal of Perinatology*.

[6] Tubbs-Cooley H. L., Pickler R. H., Meinzen-Derr J. K. (2014). Missed Oral Feeding Opportunities and Preterm Infants' Time to Achieve Full Oral Feedings and Neonatal Intensive Care Unit Discharge. *American Journal of Perinatology*.

[7] Pickler R. H., Mauck A. G., Geldmaker B. (1997). Bottle-feeding histories of preterm infants. *Journal of Obstetric, Gynecologic, and Neonatal Nursing*.

[8] Sheppard J. J., Fletcher K. R. (2007). Evidence-based interventions for breast and bottle feeding in the neonatal intensive care unit. *Seminars in Speech and Language*.

[9] Pickler R. H., Best A. M., Reyna B. A., Gutcher G., Wetzel P. A. (2006). Predictors of nutritive sucking in preterm infants. *Journal of Perinatology*.

[10] Pickler R. H., Best A. M., Reyna B. A., Wetzel P. A., Gutcher G. R. (2005). Prediction of feeding performance in preterm infants. *Newborn and Infant Nursing Reviews*.

[11] McCain G. C., Del Moral T., Duncan R. C., Fontaine J. L., Pino L. D. (2012). Transition from gavage to nipple feeding for preterm infants with bronchopulmonary dysplasia. *Nursing Research*.

[12] Korner A. F., Stevenson D. K., Kraemer H. C. (1993). Prediction of the development of low birth weight preterm infants by a new neonatal medical index.. *Journal of Developmental and Behavioral Pediatrics*.

[13] Schulz K. F., Altman D. G., Moher D. (2010). CONSORT 2010 statement: updated guidelines for reporting parallel group randomized trials. *Annals of Internal Medicine*.

[14] Dodrill P., Donovan T., Cleghorn G., McMahon S., Davies P. S. W. (2008). Attainment of early feeding milestones in preterm neonates. *Journal of Perinatology*.

[15] Wrotniak B. H., Stettler N., Medoff-Cooper B. (2009). The relationship between birth weight and feeding maturation in preterm infants. *Acta Paediatrica*.

[16] Hwang Y. S., Ma M. C., Tseng Y. M., Tsai W. H. (2013). Associations among perinatal factors and age of achievement of full oral feeding in very preterm infants. *Pediatrics and Neonatology*.

[17] Simpson C., Schanler R. J., Lau C. (2002). Early introduction of oral feeding in preterm infants. *Pediatrics*.

[18] Premji S. S., McNeil D. A., Scotland J. (2004). Regional neonatal oral feeding protocol: Changing the ethos of feeding preterm infants. *Journal of Perinatal and Neonatal Nursing*.

[19] Kirk A. T., Alder S. C., King J. D. (2007). Cue-based oral feeding clinical pathway results in earlier attainment of full oral feeding in premature infants. *Journal of Perinatology*.

[20] Ross E. S., Philbin M. K. (2011). Supporting oral feeding in fragile infants: an evidence-based method for quality bottle-feedings of preterm, ill, and fragile infants. *Journal of Perinatal and Neonatal Nursing*.

[21] Gennattasio A., Perri E. A., Baranek D., Rohan A. (2015). Oral feeding readiness assessment in premature infants. *MCN: The American Journal of Maternal/Child Nursing*.

[22] Pickler R. H., McGrath J. M., Reyna B. A. (2010). A model of neurodevelopmental risk and protection for preterm infants. *Journal of Perinatal and Neonatal Nursing*.

[23] Alperts J. R., Pickler R. H. (2012). Evolution and development of dual ingestion systems in mammals: notes on a new thesis and its clinical implications. *International Journal of Pediatrics*.

